# From the Ocean to the Lab—Assessing Iron Limitation in Cyanobacteria: An Interface Paper

**DOI:** 10.3390/microorganisms8121889

**Published:** 2020-11-29

**Authors:** Annie Vera Hunnestad, Anne Ilse Maria Vogel, Evelyn Armstrong, Maria Guadalupe Digernes, Murat Van Ardelan, Martin Frank Hohmann-Marriott

**Affiliations:** 1 Department of Chemistry, Norwegian University of Science and Technology (NTNU), 7491 Trondheim, Norway; annie.v.hunnestad@ntnu.no (A.V.H.); maria.g.digernes@ntnu.no (M.G.D.); 2 PhotoSynLab, Department of Biotechnology and Food Science, Norwegian University of Science and Technology (NTNU), 7491 Trondheim, Norway; annevogel87@gmail.com (A.I.M.V.); martin.hohmann-marriott@ntnu.no (M.F.H.-M.); 3 NIWA/University of Otago Research Centre for Oceanography, Department of Chemistry, University of Otago, 9054 Dunedin, New Zealand; earmstrong@chemistry.otago.ac.nz

**Keywords:** oceanography, marine biogeochemistry, molecular biology, cyanobacteria, iron limitation, photosynthesis, phytoplankton

## Abstract

Iron is an essential, yet scarce, nutrient in marine environments. Phytoplankton, and especially cyanobacteria, have developed a wide range of mechanisms to acquire iron and maintain their iron-rich photosynthetic machinery. Iron limitation studies often utilize either oceanographic methods to understand large scale processes, or laboratory-based, molecular experiments to identify underlying molecular mechanisms on a cellular level. Here, we aim to highlight the benefits of both approaches to encourage interdisciplinary understanding of the effects of iron limitation on cyanobacteria with a focus on avoiding pitfalls in the initial phases of collaboration. In particular, we discuss the use of trace metal clean methods in combination with sterile techniques, and the challenges faced when a new collaboration is set up to combine interdisciplinary techniques. Methods necessary for producing reliable data, such as High Resolution Inductively Coupled Plasma Mass Spectrometry (HR-ICP-MS), Flow Injection Analysis Chemiluminescence (FIA-CL), and 77K fluorescence emission spectroscopy are discussed and evaluated and a technical manual, including the preparation of the artificial seawater medium Aquil, cleaning procedures, and a sampling scheme for an iron limitation experiment is included. This paper provides a reference point for researchers to implement different techniques into interdisciplinary iron studies that span cyanobacteria physiology, molecular biology, and biogeochemistry.

## 1. Introduction

Iron is growth-limiting for phytoplankton in 25%–30% of the world’s oceans [1,2]. Since the discovery of high nutrient-low chlorophyll (HNLC) zones in the oceans, research has focused on the effect of iron limitation on oceanic organisms, especially phytoplankton. It has been estimated that phytoplankton are responsible for up to 50% of global carbon fixation by photosynthesis [3] and are the base of most aquatic food webs [4]. Recent changes in the marine ecosystem, such as increasing temperatures and acidification of the oceans caused by rising atmospheric carbon dioxide (CO_2_) have a significant impact on iron acquisition by phytoplankton, marine primary production, food webs, and biogeochemical cycling, but these impacts are still rarely investigated [5,6,7,8]. Therefore, understanding iron dynamics is essential in contextualizing any negative impact of climate change on marine primary producers.

Current research on the role of iron in the marine environment is, to a degree, split into two research fields that employ different methodologies. This includes the use of oceanographic methods to understand large-scale biogeochemical processes in which iron is playing a key role, such as primary productivity and its associated carbon export to the deep ocean [9,10,11,12,13], and laboratory-based experiments using monocultures of model organisms by employing molecular genetics to identify underlying molecular mechanisms at a cellular level [14,15,16,17,18]. Both research fields offer unique insights. Large-scale international collaborations such as the GEOTRACES program has worked wonders in bringing these research directions closer together, yet initiating smaller scale collaborations with limited infrastructure may prove a challenge despite the wealth of available data [19,20,21,22,23].

In this interface paper, we first give a brief overview of the historical perspectives of marine iron research and the role of iron in cyanobacteria such as the model organisms *Synechocystis* and *Synechococcus*. We discuss some of the biophysical, molecular biology and biogeochemical analytical techniques that can potentially complement each other, and discuss the challenges and methodological limitations encountered by using techniques from these different fields. The goal of this report is to encourage researchers from relevant fields to collaborate and foster future research initiatives that will complete our understanding of the role of iron in the oceans.

## 2. The History of Iron Limitation Research

**Figure 1:**
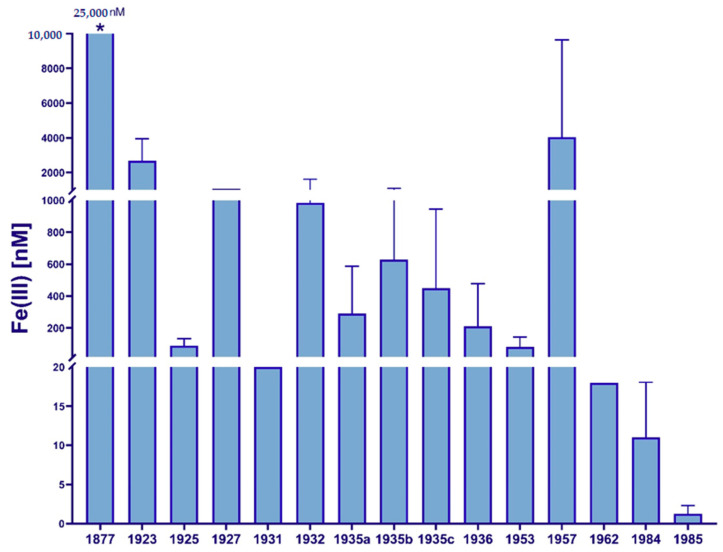
Historical overview of marine iron concentrations, as reviewed by de Baar (1994) [28]. * Proposed concentration based off data supposedly published by Schmidt in 1874 [29] and 1877 [30], as cited by Vernadsky (1924) [31] and Lewis and Goldberg (1954) [32]. Further referenced iron concentrations from Orton, 1923 [33], Harvey, 1925 [34], Wattenberg, 1927 [35], Braarud & Klem, 1931 [36], Thompson et al., 1932 [37], Cooper, 1935a [38], Thompson and Bremner, 1935b [39], Seiwell, 1935c [40], Rakestraw et al., 1936 [41], Simons et al., 1953 [42], Armstrong, 1957 [43], Menzel and Spaeth, 1962 [44], Subba Rao and Yeats, 1984 [45], and Symes and Kester, 1985 [46].

While iron was proposed to be a possible growth limiting factor as early as 1931 [24], there were no reliable methods available to corroborate this assumption. The absence of productive phytoplankton communities in HNLC zones observed in several oceanic regions remained a scientific paradox for decades. What limited the growth of phytoplankton in these regions? While iron was known to be of physiological importance due to contamination during sampling and processing, it was believed that iron concentrations in the open ocean mirrored those in terrestrial systems [25]. Patterson and Settle’s development of contamination-free laboratory methods [26] was the essential catalyst for the trace metal analysis revolution of subsequent years. For the first time, researchers were able to collect and analyze seawater samples with relatively low iron contamination levels, and it became clear that iron concentrations in the oceans were orders of magnitude lower than previously believed [27]. A historical overview of iron concentrations reported in marine samples from around the world [28] is shown in Figure 1, highlighting the discrepancies in numbers and the importance of the development of clean sampling techniques.

In 1988, John Martin proposed his “iron hypothesis” [47], supported by extensive studies both in the field and in the lab [47,48,49,50], thereby explaining the occurrence of HNLC zones. Iron had already been identified as a crucial cofactor in many enzymes, and a broader understanding of the cellular roles of iron, which are the reasons for growth limitations observed in the ocean that started to form. Several large-scale iron fertilization experiments were carried out in the following years [51,52,53], and Martin’s hypothesis on iron limitation in HNLC zones was confirmed. It would take another decade before iron was fully incorporated into biogeochemical models, and its global importance in primary production recognized [54].

## 3. The Role and Distribution of Iron in Marine Ecosystems 

Iron is involved in a wide range of important cellular functions, and can, thus, become limiting for many marine microorganisms, including cyanobacteria. For example, iron is a co-factor in the nitrogen-fixation enzyme nitrogenase. The active site of nitrogenase contains a total of seven iron atoms [55], but the enzymatic catalysis rate per unit of iron is very low compared to other iron-containing enzymes [56].

In addition, iron is involved in respiration and photosynthesis with both processes being affected by decreased iron availability [57]. The photosynthetic machinery of phytoplankton contains an estimated 80% of the organism’s total iron pool. To efficiently deal with iron deficiency, phytoplankton modify their light-harvesting systems as well as reducing the concentrations of iron-rich components within the cell [1]. One example of this is the monomerization of the PSI trimers, thereby reducing the capacity for state transitions [58]. Such modifications will allow phytoplankton to survive iron deficient conditions but do have a detrimental effect on the growth rate and photosynthetic efficiency [59].

Involvement in these vitally important cellular processes means iron most certainly can be a limiting factor for marine primary production. While iron is the fourth most abundant element in the Earth’s crust, its bioavailability for marine microorganisms is complex, which is dependent on the iron source, redox-state of the mineral and environmental factors in the marine environment [60].

### 3.1. Sources and Distribution of Iron

The primary sources of iron in the open ocean are aeolian dust, hydrothermal vents and advection [61,62,63]. While hydrothermal vents contribute to locally high iron concentrations in the benthic zones, aeolian dust is distributed over vast areas of the open ocean’s euphotic zones [1]. Coastal and near-coastal regions with high sea-ice cover receive a relatively high input of bioavailable forms of iron through ice melting [64,65], while rivers, and especially blackwater rivers, contribute to locally high inputs of dissolved iron [66]. In the Arctic (through the trans-polar drift) and Antarctic, advection from coastal zones provide important contribution of iron to open ocean areas [67,68,69].

However, the low dispersion of iron throughout the water masses leads to relatively depleted levels in large parts of the ocean [70]. In addition to low concentrations, the bioavailability of different iron forms is dependent on thermodynamic and kinetic stability of the mineral compound, organic complexation, particulate sizes, and diffusive flux, and will, thus, be largely affected by the iron source and the local environmental conditions such as pH, temperature, and redox conditions [1,71,72]. The most thermodynamically stable form of iron in oxygenated ocean waters is insoluble oxide/hydroxide-complexed Fe(III) with limited bioavailability [2]. In addition, as much as 99% of dissolved iron in seawater may occur in organic complexes [73,74] or in colloids [75,76]. These organic complexes range from abundant but diffuse, weak associations between iron and humic substances and exopolysaccharides to strong-binding siderophores as well as small organic chelators with high Fe(III)-binding affinity [77], which are secreted directly from some cells, including cyanobacteria. In general, these complexes can be distinguished by size, where an increase in molecular size is often linked to a decrease in thermodynamic stability as well as an increase in kinetic inertness, and, thus, a potential decrease in bioavailability [78]. Colloidally bound iron is generally thought to be less bioavailable, and it may also increase iron removal through aggregation into larger particles [79]. Distinguishing true soluble iron from colloidal iron fractions is an analytical challenge, and it is, thus, hard to describe how bioavailability of iron is affected by colloidization [75].

Due to this wide range of chemical interactions, uncomplexed dissolved forms of ferric (Fe(III)) iron are rare in the ocean [80]. Free or complexed Fe(III) may further be reduced to Fe(II) through photoreduction by reactive oxygen species (ROS) [81] or through bio-reduction at cell surfaces [82,83]. Fe(II) is more soluble and kinetically labile than Fe(III) and generally considered more bioavailable [84]. However, Fe(II) is oxidized to Fe(III) in the presence of O_2_ and H_2_O_2_, and the rate of oxidation increases with a growing temperature, causing a short residence time in seawater [85,86].

### 3.2. Marine Cyanobacteria in an Iron Limited World

**Figure 2:**
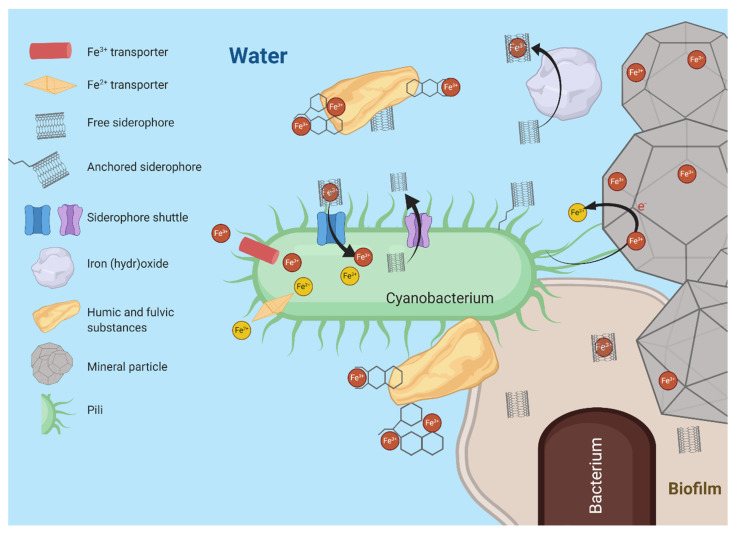
Schematic representation of marine cyanobacterial iron metabolism in relation to sources of iron. The cyanobacterium can either be free-living, represented as directly interfacing with the water (top left), or affiliated with other bacteria in a biofilm (bottom right). Iron may be available as iron oxides/hydroxides, which may be embedded in other mineral particles, or as iron complexed in humic and fulvic substances, or iron that is bound to siderophores. Free ferric and ferrous iron can be taken up by specific transport systems. Siderophores with bound iron can be taken up by the cyanobacterium with the iron released within the cell while the released siderophore is consequently re-excreted. For iron to reach the inside of the cells, two membrane systems (inner and outer membrane) have to be crossed by iron transporter proteins involved in siderophore shuttling. There are indications that a reductive step is involved in the release of iron from siderophores. A reductive mechanism has also been suggested to be active for releasing iron from extracellular iron oxides/hydroxides. Pili may aide in this process by making contact with the particles and/or directly or indirectly mediating electron transport from the cell to the iron minerals. Pili are also involved in biofilm formation, which interfaces consortia of bacteria with minerals as well as humic and fulvic components. Created with BioRender.com.

Cyanobacteria of the genera *Prochlorococcus* and *Synechococcus* dominate marine ecosystems [87]. Since cyanobacteria have a high iron demand due to their iron-rich photosynthetic machinery, they have developed an extensive range of iron stress responses, such as changing ratios of Photosystem II (PSII): Photosystem I (PSI) [88,89], decreasing iron requirements by the expression of iron-free redox proteins, e.g., IsiB [90,91], upregulation of high-affinity iron uptake transporters [92], changes in nitrogen: phosphorus ratios [93], degradation of phycobilisomes [94], and expression of the cyanobacteria-specific antenna protein IsiA [95]. Due to iron limitation and reduced bioavailability in marine environments, cyanobacteria have adapted a variety of iron acquisition strategies, as seen in Figure 2. For example, the FutABC transporter system is specific for the uptake of Fe(III) [17,96,97] and has been extensively studied in cyanobacteria. Acquisition of Fe(II), though rapidly oxidized in the euphotic ecosystem, is typically performed by the high affinity FeoAB transporter [17]. Many marine cyanobacterial genomes also encode for siderophores, but the use of siderophore-mediated iron acquisition methods in cyanobacteria has been highly discussed in recent years [98]. Recent research proposed type IV pili facilitated reduction of external electron acceptors such as ferric oxides by cyanobacteria, thereby increasing iron bioavailability [99,100]. It is possible marine cyanobacteria have more iron acquisition mechanisms that have yet to be characterized.

## 4. From the Ocean to the Lab 

To understand iron limitation, researchers often focus on analyzing iron composition in situ. However, analyzing biological samples for ocean-relevant iron concentration without contamination still poses major challenges. Many oceanographical and biogeochemical parameters can be measured either through the use of autonomous vehicles or in situ at sample sites [101], but the redox chemistry and potential for iron contamination have made the application of such analytical tools for iron determination challenging [102,103]. Autonomous vehicles and automated samplers often contain metal parts, which can easily contaminate samples, and rapid oxidation of Fe(II) at temperatures above 15 °C [85] in an oxic environment makes analysis of separate iron redox states difficult. Shipboard methods for analysis of Fe(II), Fe(III), and total iron are available but require careful sample handling and rigorous clean techniques [19,20,104,105].

In addition to monitoring iron concentrations and speciation in the oceans, it is important to understand how photosynthetic communities react to changes in iron concentration and speciation. One of the longest used proxies for phytoplankton behavior is the measurement of biomass using chlorophyll *a* analysis [106,107,108]. However, chlorophyll *a* might be an unreliable proxy as its concentration can differ greatly independently of biomass. The chlorophyll *a* cell content varies between species, and as a result of a cell’s response to environmental parameters, such as the light level and nutrient limitation [109]. In situ measurements give insights into the condition of photosynthetic communities, but the various methods for analysis of chlorophyll *a* still cause disparity in results due to differences in detection methods (e.g., fluorometry and spectrophotometry) as well as interference from degradation products and other pigments [110,111]. Widely used satellite imaging only penetrates to approximately one-meter depth, and, therefore, photosynthetic communities living below this depth are not assessed by this technique. Since cyanobacteria and other phytoplankton can thrive at much lower depths, this may contribute to large underestimations of biomass. Satellites only deliver trustworthy data on a relatively clear day, and, when no ice cover is present, this technique limits estimations of biomass [106].

Natural variations such as the timing of blooms, mean in situ experiments are difficult to carry out in the open ocean, and make multiple driver experiments a major challenge [112]. It is extremely demanding to monitor all relevant parameters for assessing the physiological state of phytoplankton and chemical composition of ocean water in situ. Multiple driver scenarios are often more reliable in a laboratory setting due to controlled conditions.

**Figure 3:**
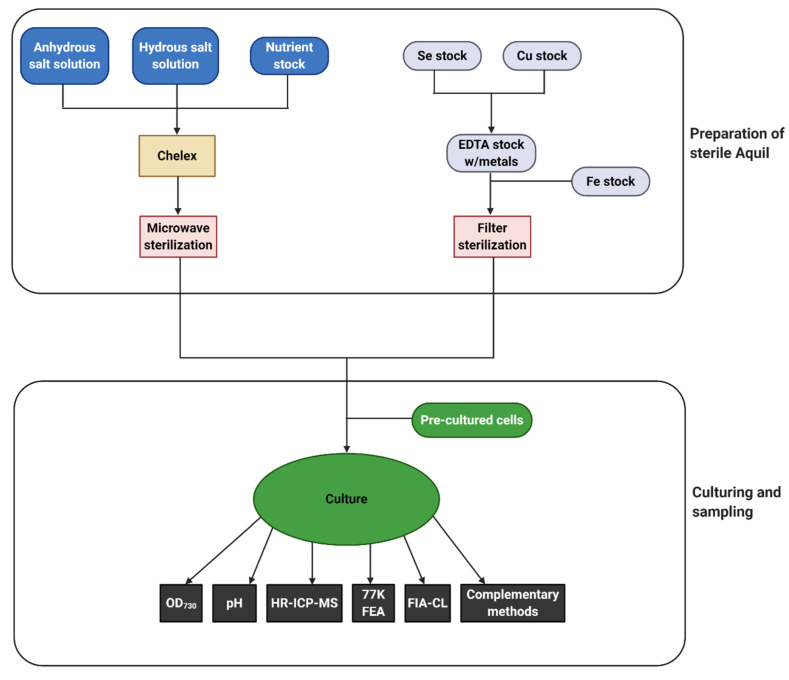
Workflow for a typical iron limitation experiment using Aquil. The workflow is split into two distinctive parts. Preparation of sterile and trace-metal free Aquil includes the preparation of several stock solutions for, e.g., copper and selenium as individual metal stocks, before sterilization and subsequent addition to a final medium. Daily sampling of biological parameters such as the OD_730_, pH, and 77K Fluorescence emission analysis (indicated as 77K FEA) are strongly recommended. Inductively coupled plasma mass spectrometry (indicated as HR-ICP-MS) and Flow Injection Analysis (indicated as FIA-CL) should be sampled according to a carefully considered sampling scheme, taking into consideration the length of the experiment as well as the density and health of the culture. An example of a full sampling procedure including all protocols can be found in the Supplementary Materials.

To elucidate the response of cyanobacteria to iron limitation and understand the role of iron in marine ecosystems, it is necessary to establish reliable techniques for following iron concentration and iron fractions over time and to simultaneously characterize the changes in internal iron concentration and the physiology of the affected cyanobacteria. Systems modelled on the natural environment in the laboratory are necessary to control all experimental parameters. The use of a well-defined growth medium, such as the synthetic seawater medium Aquil [113], is essential for iron limitation studies [22]. The composition of Aquil can be adapted to the study organism, e.g., by adding or removing nutrients, or changing nutrient concentrations in order to avoid an unwanted stressor influence. In the following sections, we will describe individual molecular biology, spectroscopy, and analytical chemistry techniques and how they can be combined to study iron acquisition and physiology of cyanobacteria. A simplified typical workflow scheme for a simple culture study is shown in Figure 3.

### Size Fractions of Iron in Seawater 

**Figure 4:**
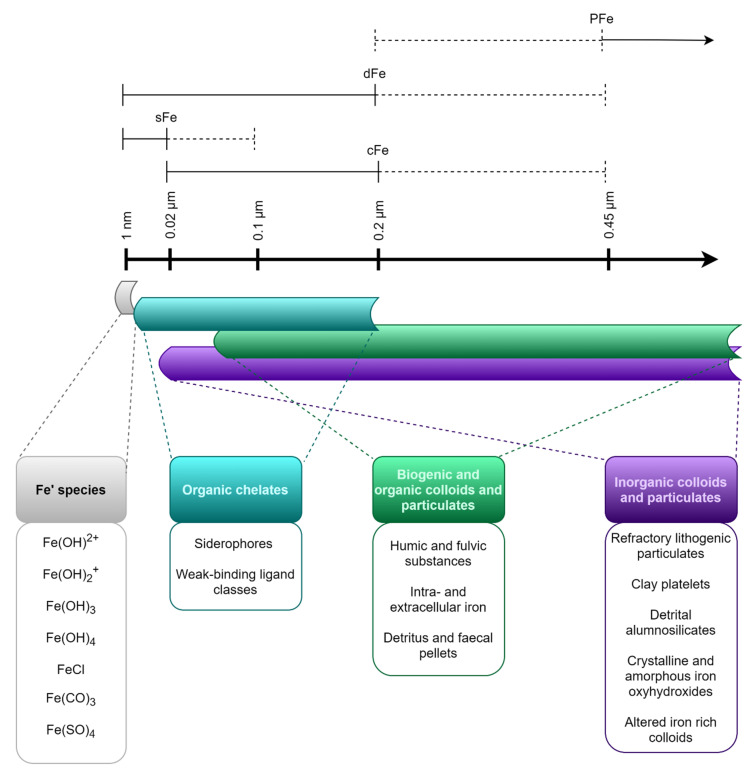
The operationally defined size fractions of iron in the ocean. Sizes of particulate iron (PFe), dissolved iron (dFe), colloidal iron (cFe), and soluble iron (sFe) are indicated in µm. Dotted lines indicate the range of definition. Typical iron-containing substances found within each size spectrum are indicated below. Modified from Bruland and Rue [115] as well as von der Heyden and Roychoudhury [114].

During iron studies in the lab or natural systems, the size fractions of the element are often given careful consideration as different iron pools. The size fractions are operationally defined by size filtration. Since these fractions are operationally and somewhat arbitrarily defined, they give little information about the structure and nature of the iron-bearing compounds found within each fraction [54], and some types of particles and molecules may be found in several different fractions [114]. Figure 4 gives an overview of common size fractions, and the types of compounds included in each fraction.

Total dissolvable iron (TFe) is defined as the concentration of available iron, which can be detected in an unfiltered sample after acidification and long-term storage, and incorporates all other fractions [20]. Dissolved iron (dFe) is the fraction of iron able to pass through a filter with pore size <0.2 or 0.45 µm (operationally defined and varies from study to study), while particulate iron (PFe) cannot pass through the same filter [75]. The dissolved fraction can be further divided into colloidal (cFe, 0.02 µm-0.2 µm) and soluble (sFe, < 0.02 µm) fractions [114].

TFe in a natural sample includes all lithogenic particles and microbial cells in addition to dissolved iron forms, so it only gives limited information about biologically relevant iron concentrations. To gain a more comprehensive picture of iron availability, other fractions should be sampled simultaneously. In natural samples, PFe will often be a combination of lithogenic particles and biological material such as faecal pellets and microbial cells. The fate of these particles is determined by their size, density, and interaction with other particles, and they typically sink rapidly, limiting the associated iron’s availability to primary producers [116]. However, there is evidence of more dynamic particulate iron cycling in certain areas. For example, Frew et al. (2006) observed efficient conversion of lithogenic PFe to biogenic PFe in the upper water column of subantarctic waters. This can lead to further changes of biogenic PFe through grazing and cell lysis, producing smaller-sized compound with a potential for a higher degree of bioavailability [117]. dFe, in contrast to PFe, often displays a nutrient-like profile in the open ocean, indicating scavenging and biological utilization in the euphotic zone, and remineralization at depth [118]. Only more recently has focus been placed on studying colloidal and soluble iron separately within the dFe pool [114], producing increasingly detailed information about the distribution and significance of these iron fractions on a global scale.

In a laboratory setting, the particulate iron fraction can be used as a proxy for material bound to and encapsulated by cyanobacteria or other phytoplankton in an axenic culture [119]. Determining intracellular and extracellular iron concentrations is possible by washing cells to remove surface bound iron [120] and then measuring iron in both fractions. Changes in dFe(III) concentrations should correlate with changes in PFe(III) concentrations in a culture, providing information on uptake and potential solubilization of iron over time. Monitoring changes in dissolved Fe(II) concentrations in parallel may also give more information about the fate of iron within the culture. TFe concentration can be used to monitor iron contamination of a lab culture. Detecting any changes in the total iron concentration in the culture is important to safeguard the validity of results produced.

## 5. Challenges and Methodical Problems in Iron Limitation Studies 

Next, we will focus on some of the techniques that allow studying iron limitation in cyanobacteria. Though useful, all methods have drawbacks and limitations. In this section, we discuss specific challenges and compromises required when working with cyanobacteria in laboratory-based iron limitation studies.

### 5.1. Physiological Challenges 

Ideally, when investigating iron limitation in the laboratory, a study should mimic the natural environment of an organism as closely as possible, while changing only the parameters of interest, i.e., iron concentrations and speciation. Other potential drivers/stressors must be excluded to ensure that the observed phenotype is caused by iron limitation and not by, e.g., temperature, macronutrients, or high-light stress. However, growth conditions of laboratory strains are often optimized, which may result in growth conditions not found in nature. On the other hand, these artificial growth conditions enable identification of the underlying iron-related phenotypes and iron-related stress coping mechanisms without the interference of unexpected or unknown factors not related to iron stress.

An important methodological challenge, which creates the need for compromise, is the choice of temperature. Researchers may prefer a higher incubation temperature for faster growth of cyanobacterial strains commonly used in laboratory research, such as *Synechococcus* sp. PCC 7002 or *Synechocystis* sp. PCC 6803, as this results in shorter experimental times. The measurement of Fe (II), however, requires lower temperatures to avoid oxidation. While Fe(II) may still be present in the medium at a higher temperature, analytical systems may not be able to detect it before its oxidation to Fe(III) within the system itself. Additionally, such high temperature-optimized growth rates are rarely found in nature, making it harder to extrapolate results to natural systems. Finding a temperature at which growth can be achieved without cold stress, but with manageable growth rates while still meeting detection limits for Fe(II) measurements, is, therefore, a challenge. The influence of temperature, as well as the photoreduction of Fe (III) [121,122], highlights the importance of an abiotic control to separate physical and chemical iron transformations from those that are biologically mediated.

When working in controlled laboratory conditions, experimental strain selection must be critically assessed. Only a few marine cyanobacterial strains have been cultured in laboratories, they are typically fast-growing, often cultured in optimal, not necessarily natural, growth conditions [123,124]. Once established, model organisms are often re-cultured for many years in laboratory research. Constant re-culturing of strains results in organisms adapting to laboratory conditions, such as a constant light or constant temperature regimes, which consequently results in epigenetic and/or genetic changes in the genome of the model organisms. A well-known example is *Synechocystis* sp. PCC 6803, which has been used as a cyanobacteria model organism for about 50 years [125,126]. The strain contains, among other genetic changes, a frameshift mutation in *slr0162*, a gene encoding for PilC, which leads to the abolishment of twitching motility [127] and changes in biofilm formation [128] that may impact utilization of iron. Undeniably, adaption is a natural process and cannot be avoided in the laboratory. However, it is important to note that laboratory-specific mutations and adaptions might influence phenotypes when comparing results from different research groups.

### 5.2. Iron Related Challenges

When conducting iron limitation studies with axenic cultures, such as those presented in this interface paper, both sterile and iron-free conditions are essential. However, working under both these conditions imposes restrictions on the experimental setup. Traditional sterile techniques such as autoclaving introduce iron contamination, and alternative solutions are required such as microwaving the base seawater of a medium [129], while filter-sterilizing metal and nutrient stocks separately to avoid any temperature damage. Ethanol for surface sterilization purposes should be used sparingly as it may introduce iron contamination. This potential for contamination exists for all reagents and equipment with unknown iron concentrations [22]. A method to overcome the necessity for stringent ethanol use is the implementation of closed systems with automated sampling. Such systems reduce the risk of both biological and trace metal contamination of cultures, and leave researchers free to treat subsamples according to the type (biological or trace metal) of further analysis. All manipulations should be carried out in a laminar flow hood, and a class 100 cleanroom should be used if available when working with low iron concentrations. However, these rooms are expensive to set up, and not all research groups have access to suitable locations. When working with iron concentrations above picomolar levels, it is possible to create a makeshift clean lab with a plastic coating on the walls and benches, as well as cleaning all surfaces with ultrapure acids. Whether in a class 100 cleanroom or in a makeshift clean lab, all researchers should use microporous laminated clean suits (Tyvek^®^, DuPont, Wilmington, DE, USA), shoe covers, and hair nets. Mouth covers may also be necessary, and no metal pipes, chairs, etc. should be present inside the clean area. All plastic consumables should be cleaned according to stringent procedures [19], and every step of the experimental setup needs to be controlled for bacterial and trace metal contamination. This includes the use of HEPA-filters when bubbling air through cultures as well as closed culture systems with permanent sampling systems. When using such a cost-efficient clean room, the use of suitable blanks, contamination controls, and following the total iron concentration becomes even more important than when a standard cleanroom is used.

The choice of iron source and concentration is also critical when conducting iron limitation experiments. For example, iron stocks cannot be purified using Chelex or other chelators, and it is important that all chemicals are of a trace metal grade. Bioavailability, possible chemical changes, and complexation all need to be considered when evaluating iron sources, and final choices must reflect the specific research questions. If the iron source itself is of interest, then an experiment can be set up using different sources at different concentrations. An example of this would be designing an experiment around iron sources of varying solubility, such as the minerals hematite and goethite, and the amorphous FeCl_3_. It is important to note that many minerals have such low solubilities, that even creating stock solutions is a challenge. This can be overcome by adding iron in a solid form, or by using acidified stocks [22]. Interactions between any added EDTA and the iron source should also be considered. If an experiment is mimicking the natural conditions of a specific region, it is important to consider which iron forms are most prevalent in that study area. In either case, baseline experiments should be set up using an iron concentration that does not stress the organism in question. This is especially important in the study of genetically modified strains, as it will provide a baseline for response in iron deplete conditions.

Due to the transient nature of iron chemistry, a stable and representative sample is an integral component of iron speciation analysis. Temperature greatly reduces the half-life of Fe(II) above ~10 °C [121,130,131]. Differences in pH also affects the rate of oxidation. At pH levels below ~4, Fe(II) is the dominant species and oxidation rates are independent of pH, while $\mathrm{Fe}{\left(\mathrm{OH}\right)}_{2}^{0}$ concentration affects the rate of oxidation at pH between ~5 and 8 [132,133,134]. In addition, organic matter such as iron-binding ligands form complexes with dissolved iron. Thus, organic matter likely affects iron solubility and bioavailability [78,135,136], and can be analyzed using stripping voltammetry [137,138] and high-performance liquid chromatography (HPLC). Moreover, photo-reductive processes also play an important role in the reduction of iron [139,140]. Consideration of parameters such as light, organic matter, pH, and temperature are imperative for a comprehensive approach to iron speciation studies.

Controls in this type of study should help elucidate which changes are biologically mediated and which are purely chemical/physical. An abiotic control should, thus, be set up with identical medium composition, and the same exposure to physical and chemical parameters, such as light, temperature, and air bubbling. The sampling scheme should be identical for cultures and controls.

## 6. Methodology

Working with iron in a multidisciplinary context allows for the use of a wide array of methods, and the collection of data from a large set of parameters. In this setting, it is important to plan carefully in order to include as many of these essential parameters as possible while avoiding producing redundant data. To achieve this, molecular biologists and marine biogeochemists must determine which methodological parameters complement each other, and which methods from the separate fields may yield the best data. In this section, we hope that the molecular biologist will find the subsections on High Resolution Inductively Coupled Plasma (HR-ICP-MS) and Flow Injection Analysis Chemiluminescence (FIA-CL) especially illuminating, while marine biogeochemists might find new useful information in the subsections on 77K Fluorescence analysis (FEA), spectroscopic approaches, and molecular biology approaches.

With the complexity of a natural system comes the increased importance of robust methods and systematic tests and controls ensuring the quality of data produced. All methods should be validated through stringent testing and use of controls, such as the analysis of certified seawater reference materials (e.g., SAFe [19], CASS, and NASS [141]), and use of external, validated laboratories for replicate analysis of and intercalibration of samples. Such testing and validation ensure quality in analysis and strength in data produced. It also helps ensure uniformity in methodology, minimizing the risk of analytical errors impacting the conclusions of scientific studies. This focus on analytical quality will ensure traceability [19].

### 6.1. Analysis of Fe(III) Fractions by HR-ICP-MS

High resolution inductively coupled plasma mass spectrometry (HR-ICP-MS) can be used to quantify the distribution of iron in different fractions [20], such as total (or total dissolvable, TFe), dissolved (dFe), and particulate (PFe) in seawater or growth medium [114,142,143]. Such fractions are mainly operational but provide information about how iron is being transformed by cyanobacteria and other marine microorganisms. For samples containing cyanobacteria or other microorganisms, an intracellular fraction, or cellular iron quota, can be obtained after washing cells with a chelator such as oxalate to remove iron adhering to the cell surface [120]. This quota can be used to assess adaptations by cells to reduce cellular iron content when iron supply is not abundant. Obtaining intracellular concentrations requires stringent purification of reagents and careful sample handling and should be done in addition to measuring PFe concentrations. HR-ICP-MS is not able to provide information on the redox-state of iron, since Fe(II) and Fe(III) will both be part of the same signal, unless the two fractions are separated prior to analysis.

The sample medium (matrix) can affect the detection of elements of interest when running HR-ICP-MS analysis. Iron analysis, in a seawater matrix, is a challenge due to the element’s low concentration as well as the substantial interference of other elements (e.g., Na, K, Cl) [144]. Interferences range from spectral interference by polyatomic species to non-spectral interference by accumulation of salt on the cones and electrostatic lenses of the instrument, causing a signal drift. Issues stemming from the matrix can often be resolved by diluting the sample. However, when working with seawater samples, instrument sensitivity may not be adequate for the resulting low iron concentrations [145].

A combined solution to the problems of low iron concentration and matrix interference is pre-concentration with a chelating resin, where interfering matrix elements can be removed by rinsing before iron and other trace elements are eluted from the resin and detected by HR-ICP-MS [144]. This type of pre-concentration can be done either offline or online. However, the online option requires additional instrumentation, such as the SeaFAST system (Elemental Scientific Inc.). Since manual sample handling is minimal, performing this step as an online part of the HR-ICP-MS workflow results in reduced risk of contamination [145]. Methods using isotope dilution with standard additions have also been developed, allowing the simultaneous detection of several trace elements at seawater concentrations [142].

Synthetic seawater media are generally prepared using EDTA as a metal ion buffer, regulating the formation of insoluble precipitates and, thus, increasing metal availability while simultaneously keeping free ionic metal concentrations at nontoxic levels [22]. However, since EDTA is a strong chelator, it will compete with most pre-concentration resins and, thus, interfere with recovery of metal species in the sample. This can be avoided by breaking the EDTA complexes either by treating samples with ultraviolet (UV) radiation [146] by sufficiently lowering the sample pH over a long time period [147], or by a combined lowering of pH and microwave treatment [148]. Following treatment, the sample should be brought back to the appropriate pH for optimal resin performance without introducing any contaminants.

Samples for HR-ICP-MS should be collected, at least, in triplicate, with the sample size being dependent on whether pre-concentration is needed. Samples should be acidified to pH < 2 using Ultrapure (UP) grade HCl or HNO_3_ and stored using trace level clean procedures until analysis [19]. Particulate and intracellular samples should be stored on folded filters in trace-metal cleaned plastic petri dishes. Such samples need to be digested before analysis, which can be done using ultra-pure HNO_3_ digestion in an Ultra Clave system. An example of a full sampling procedure is described in the Supplementary Materials.

### 6.2. Measurement of Fe(II) 

Flow injection analysis (FIA) is generally defined as liquid sample solutions eluted into an unsegmented liquid flow stream with subsequent treatment or analysis of the analyte [149,150]. FIA followed by chemiluminescence (FIA-CL) Fe(II) detection has been a widely employed method in marine iron studies [131,151,152,153,154]. The FIA-CL method can measure Fe(II) at naturally occurring nanomolar and sub-nanomolar concentrations using a luminol-based reagent with no chemical pre-treatment of samples. However, pre-FIA analysis sample filtration is necessary to avoid biological contamination of the FIA system. FIA-CL is an inexpensive and highly sensitive method for measuring Fe(II) concentrations continuously or discretely, both shipboard and in the laboratory. This method is particularly useful during iron limitations studies as it can be used to monitor the presence of Fe(II) in growth media such as Aquil. Analysis of this data can, for example, give insight into the external reduction of Fe(III) by cyanobacteria.

In addition to the sample considerations mentioned in Section 5.2, parameters within the FIA-CL system require optimization to achieve the best detection limits and narrower peaks [152]. Optimal instrumental parameters such as detector sensitivity and chemical ratios between reagents and reactants have been reported in different publications [131,155,156]. However, different sample materials and instrumentation require individual optimization. Studies investigating uncertainties in the FIA-CL calibration curves have suggested that the replacement of peristaltic pumps with micro-sequential injection piston pumps [157,158,159] improves the reproducibility and sensitivity of the analysis.

FIA-CL analysis of Fe(II) can also be done with sample pre-treatment through a pre-concentration column [155,160] when Fe(II) concentrations are low, or conditions favor rapid oxidation to Fe(III). Such pre-concentration can be performed using various chelating resins with a number of different functional groups, such as ferrozine, 2-nitroso-5-(*N*-propyl-*N*-sulfopropylamino) phenol, and brilliant sulfoflavine [160]. However, the FIA-CL method with luminol can resolve sub-nanomolar concentrations without chemical treatment of samples or pre-concentration columns and is often preferred due to its relative simplicity. Different Fe(II) flow injection methods with and without preconcentration have been compared and shown to have inconsistencies across methods [81], highlighting the need for standardizing an Fe(II) procedure for comparison of data.

Additional chemical analyses by FIA, such as hydrogen peroxide analysis, can aid in understanding the mobility of iron. During the oxidation-reduction reaction of Fe (II)/Fe(III), hydrogen peroxide is formed as a byproduct and can, thereby, chemically oxidize Fe(II) [86]. Thus, parallel measurements of hydrogen peroxide and Fe (II)/Fe(III) by FIA systems can be used to gain more information about Fe(II) and Fe(III) redox chemistry [132].

Another notable method for measuring Fe(II) is spectrophotometric analysis after ferrozine complexation [161,162]. Ferrozine selectively binds Fe(II), and the complex may be pre-concentrated by passing the sample through a column containing a chelating resin. The complex is then eluted with methanol and detected by a spectrophotometer. This method avoids rapid Fe (II) oxidation caused by temperature, oxygen, and other oxidants due to the instant formation of an Fe(II)-ferrozine complex [163]. Developments of the ferrozine method includes the creation of a microfluidics sensor, where Fe(II) can be measured continuously with a deployed instrument. An added reduction step will allow for the same sensor to also measure total Fe [164]. Increasing miniaturization and automatization of certain environmental monitoring methods, such as in the field of microfluidics, provides great potential for the future [164,165,166].

### 6.3. Assessment of Photosynthetic Properties 

The analysis of pigments such as chlorophyll *a* can be used in a variety of ways to estimate the state of the photosynthetic machinery [167,168,169], which can, in turn, be related to iron limitation. Some of these methods are clearly more relevant in an oceanographic setting, such as the satellite-based estimation of Chlorophyll *a* in seawater. We present here two of the methods that will provide the largest amount of detail regarding photosynthetic properties in an axenically grown culture.

A fundamental method in photosynthesis research is the assessment of photosynthetic pigments, such as chlorophylls and carotenoids by extraction with a non-polar solvent. This analysis can be the starting point to understand stress conditions. Under most stress conditions, the relative amount of chlorophyll per cell decreases, while the number of carotenoids increases. When the extracted pigments are analyzed further using chromatographic separating techniques, such as high-performance liquid chromatography, more detailed physiological insights can be obtained. Chromatographic techniques also allow identification of the composition of photosynthetic consortia by identifying group-specific pigments [170,171].

To connect changes in iron concentration with changes in cyanobacteria physiology, fluorescence emission analysis at 77K (77K FEA) can be used. This method was recently reviewed by Lamb et al. [172]. During 77K FEA analysis, a defined number of cells are excited by a specific wavelength of light, and the emission spectra measured with a spectrometer. The emission spectra give insight into the organization of the photosynthetic machinery as different complexes emit fluorescence at different wavelengths. Excitation at 440 nm targets chlorophyll associated with PSI and PSII while 580 nm mainly excites phycobilins contained in phycobilisomes that are mainly associated with PSII. PSII-associated chlorophyll typically has emission peaks at 685 and 695 nm [173] while PSI-associated chlorophyll emits between 720 and 740 nm [174]. The low-temperature during excitation and emission prevents intramolecular vibrations, causing a sharpening of measured chlorophyll fluorescence emission peaks and enabling emission measurement of PSI-associated chlorophyll. Shifts in peak emissions can indicate physiological stress such as nutrient limitation stress, which leads to decoupling and reorganization of antenna systems from the photosystems.

Fluorescence spectroscopy at 77K requires only small sample volumes of around 1 mL when chlorophyll *a* concentration is ca. 2 µg/mL. However, since phytoplankton concentrations can be very low in extreme environments, natural samples often require pre-concentration by centrifugation. Laboratory-based cultures are usually higher in chlorophyll concentration than seawater and can be used unconcentrated or may even need to be diluted. Due to the low sample volume, the relatively easy setup, and low cost of the equipment, 77K FEA can be deployed on research vessels and used in small laboratories [175].

A hallmark protein for iron limitation is the iron stress-inducible chlorophyll *a*-containing, protective protein IsiA. This protein has a fluorescence emission peak at 685 nm. Due to the accumulation of IsiA around PSI during iron stress [95], the presence of IsiA can be identified by 77K FEA as an increase in the 685 nm and 720 nm fluorescence emission. The expression of the protective IsiA protein also affects the light absorption spectra of whole cells. Accumulation of IsiA causes a shift of the 685 nm absorption peak to lower wavelengths in the whole cell absorption spectra [94]. Another change to the whole cell absorption spectra is due to the reduction of phycobilisomes during iron stress, where phycobilin absorbance around 630 nm is reduced [94].

Chlorophyll fluorescence emission at 77K as an indicator of iron limitation should always be evaluated with caution as other forms of stress can result in complex physiological responses with similar fluorescence spectra to iron limitation. In general, the spectra can be a useful method for qualitative analysis, but it is not suitable for quantitative analysis. For example, at lower PSII levels, chlorophyll fluorescence emission at 77K from PSI contributes increasingly to the peak at 695 nm, which is usually assigned to PSII, which then influences the calculated PSI:PSII ratio [176].

Photosynthetic machinery and its efficiency can also be assessed through quenching analyses, using pulse amplitude modulated (PAM) and fast repetition rate fluorometry (FRRf) [177]. These techniques can assess photosynthetic efficiency through a relative electron transport rate [122]. Additional quenching parameters, such as sigma (σ) and tau, (τ) give more information on the state of the photosynthetic system. The σ parameter gives the effective cross section of PSII light harvesting antenna, measured in nm^−2^, and provides a parameter for the efficiency of energy delivery within the photosynthetic system. The τ parameter represents the turnover time of PSII and is a measure of how fast PSII is re-oxidized after reduction. These parameters change in environmental stress conditions, such as an iron limitation, and can indicate pigment groups or taxa present in natural samples [123]. Fluorescence quenching analysis can be used to indicate status of photo-acclimation of phytoplankton including photo-inhibition [124].

In addition to methods that analyze chlorophyll fluorescence, there are several complementary methods available that assess the efficiency of photosynthesis. Examples include the measurement of a photosynthetic oxygen production rate through ^18^O dilution [126] and approximation of photosynthesis through ^14^C uptake [127]. Details of these methods go beyond the scope of this report.

### 6.4. Assessment of Cell Number and Features

Standard techniques, such as spectrophotometry at 730 nm (OD_730_), can be used to efficiently measure an increase in the cell number over time. Fluorescent microscopy techniques and flow cytometry can be used to asses changes in the cell size over time. Flow cytometry was instrumental in identifying cyanobacteria belonging to the *Synechococcus* and *Prochlorococcus* genera in the oceans [178]. In addition to identification and enumeration of these picocyanobacteria, flow cytometry can provide additional information on cell chlorophyll concentration (via fluorescence) and cell complexity [179]. The latter parameter can be obtained by the ability of (cyanobacterial) cells to scatter light. The greater the concentration of fine-structural components, such as thylakoid membranes and glycogen granules present in a cell, the higher the proportion of light scattered by that cell is.

### 6.5. Molecular Biological Approaches

In order to elucidate the physiological response to iron limitation, it is crucial to understand the molecular mechanisms and, therefore, the underlying genes encoding the different iron-associated proteins and to identify new, yet unannotated, genes. For this, techniques used in the area of molecular biological can be used to study and, thereby, elucidate the effects of iron limitation on cyanobacteria. For example, deleting or replacing a gene of interest is commonly used to identify genes involved in iron homeostasis or iron stress. A complete overview of useful techniques is out of the scope of this interface paper. However, an excellent review of the challenges of developing genetic tools for cyanobacteria and an overview that details current methods has been published by Berla et al. (2013) [180]. The following section is meant as a short inspirational section for further research design and focuses only on the field of functional genomics. Other omics disciplines can be useful to deepen the possibilities of iron limitation research, but are not taken into account in this paper.

Modification of genes of interest should be an essential part of iron stress experiments if the goal of the experiment is identifying underlying genetic mechanisms involved in iron stress. Once a gene of interest is deleted, modified, or silenced, the model organisms can be investigated under different conditions such as under iron stress inducing and non-iron stress inducing conditions [181,182,183]. Comparing the wildtype to the strain containing the mutation with regard to phenotype and the previously discussed methodology can give insight into the role of the gene in iron stress responses. This research design is already widely adapted in molecular research.

Typically, model organisms are used when working with molecular biological approaches and many tools have been developed to aid researchers in changing and adapting the genome of the model organisms. For example, for the marine cyanobacterium *Synechococcus* sp. PCC 7002, various protocols are available for plasmid-based transformations or natural transformations by homologous recombination. Both techniques are commonly used with cyanobacteria [184,185,186,187]. Advantages in high-throughput genetic methods, such as the generation and screening of mutant libraries, may help the annotation of gene function, and, thereby, greatly aid grouping of genes involved in stress responses, such as iron shortage, in cyanobacteria [188,189]. Additionally, the steadily decreasing costs of synthesizing DNA enables the assembly of complex DNA constructs that can be transferred to the organism of interest, enabling the deletion of genes of interest. Although genetic deletion is still a time-consuming process, and off-target transformations remain a problem, in recent years, progress has been made to improve transformation times and target specificity [185,190].

Recent advantages with the use of CRISPR-Cas systems in cyanobacteria [191], promise fast and effective generation of strains carrying multiple modifications.

Changes in gene expression, as a consequence of varying environmental conditions such as iron availability, can be assessed by sequencing the total RNA content to identify the abundance of mRNA that indicate gene expression. For this, both wildtype and modified strains can be used, dependent on the hypothesis of the research. RNA sequencing techniques (RNAseq) have already been widely employed for cyanobacteria grown under different iron conditions, enabling the identification of genes involved in iron homeostasis [192]. Dependent on the isolation and sequencing methods chosen, it is possible to identify non-coding RNAs, thereby, enabling researchers to investigate the regulation of iron homeostasis and direct and indirect interactions of iron homeostasis pathways. For these studies, cells of wildtype strains are typically grown under a standard reference condition and a stress condition in at least triplicate to enable statistical comparison. A high concentration of cells guarantees the collection of sufficient genetic material. Stress cells and reference cells are snap-frozen after harvesting to prevent changes caused by the collection method. Having a reference genome available is desirable to map the RNA sequencing results. Recently, sequencing costs have decreased dramatically. Therefore, sequencing the genome of a novel cyanobacteria has become more affordable.

Microarrays, on the other hand, are more cost-effective and have been used extensively to study iron limitation and other stress conditions in cyanobacteria in the past [89]. A wide variety of datasets are already available [193]. However, analysis can only be done for pre-defined sequences and genes that are expressed at a low level are often not detected by microarrays [194]. This limits the application of this otherwise useful technique when working with organisms not yet fully annotated or when attempting to identify novel gene functions or quantify gene expression.

Establishment of new sequencing methods such as nanopore sequencing is rapidly advancing with regard to costs and accessibility so that there is a chance that this review will already be outdated with regards to the functional genomic overview. Additionally, bioinformatics tools such as the Galaxy platform [195] enables even researchers without extensive training to effectively analyze this data [196]. This development enables laboratories with different budgets and expertise to participate in functional genomics research, thereby increasing the possibilities of an elaborated research design.

### 6.6. Complementary Methods 

Several techniques that allow detailed insights into iron metabolism of cyanobacteria exist. We mention three complementary techniques here, which we believe provide particularly valuable data when studying the uptake and distribution of iron in a cyanobacterial culture. However, a multitude of other methods exists, which may provide novel insights. An example includes the monitoring of the exchange of gases such as oxygen (O_2_), carbon dioxide (CO_2_) [197], and nitrogen (N_2_) [198] in order to quantify rates of photosynthesis, and of nitrogen fixation in nitrogen fixing cyanobacteria.

#### 6.6.1. Measuring Iron Uptake Rates by Radioactive Iron

The radioactive isotopes of iron, known as iron-55, and iron-59, can be used to measure uptake rates by cyanobacteria [199,200], which can be further used to quantify the bioavailability of different Fe-substrates as well as probing iron uptake mechanisms involved [82].

Iron-59 is a β and γ emitter, has a half-life of 44.6 days, and a decay product of Cobalt-59. Iron-55, which is the more commonly used isotope, has a half-life of 2.73 years and decays by electron capture to Manganese-55 releasing Auger electrons and X-rays [201]. Prior to the use of any radioactive compound, it is advisable to contact the Institution Health and Safety Officer so that all institutional regulations regarding use of radioactive compounds can be followed [202]. In addition, the isotope supplier usually has informative user guidelines (e.g., Perkin Elmer [203]).

To measure iron uptake rates, radioactive iron (FeCl_3_) is added at tracer levels to culture medium, usually forming ca. 10% of the total iron [204]. Cells from an acclimated, exponentially growing culture are re-suspended in the fresh, labeled medium. During the incubation period (4–6 h), subsamples are removed, and the cells collected on filters, which are then flooded with a rinse solution such as Ti citrate [205] or oxalate-EDTA [120], to remove any extracellularly bound iron before being rinsed with filtered seawater or NaCl solution. Iron-isotope uptake is measured with a scintillation counter along with that on appropriate filter blanks. The total iron uptake at each time point can be calculated using the iron-isotope: unlabeled iron ratio and the rate calculated from these results. To measure ferric iron uptake, ferrozine can be added since this chelates Fe(II) preventing its uptake [206]. Ferrous iron uptake can be measured in the presence of ascorbate, which reduces Fe(III) to Fe(II) [207]. Uptake rate experiments can also be performed with iron-55 chelated to various ligands such as EDTA or siderophores, e.g., deferoxamine B and aerobactin, to determine if cyanobacteria can access iron from these compounds [97,208,209,210]. These experiments may need to be incubated in the dark, depending on the chelator, to prevent any photoreduction of the iron-55 ligand complex [211].

#### 6.6.2. Stable Isotopes

Many elements exist in nature in more than one isotopic form. Stable isotope analysis of macronutrients, such as C and N, has provided valuable information about the growth conditions of cyanobacteria [212], the source of cyanobacterial nutrients [213], and even interactions between symbiotic organisms [214]. However, the technique is now increasingly being used for micronutrients.

Analysis of stable isotopes and their ratios, measured by Multicollector Inductively Coupled Plasma Mass Spectrometry (MC-ICPMS), has demonstrated that heavier isotopes of iron are preferentially taken up by a range of microbes, bacteria [215], phytoplankton [216], and cyanobacteria [217]. Measurements of a range of trace metals is possible from the one sample.

Advances in the preconcentration of trace metals in seawater have permitted measurement of the concentrations of Fe stable isotopes [218,219], even at the low concentrations found in the subantarctic, as well as other trace metals such as cadmium and zinc. Instrumentation for preconcentration, such as the seaFAST system (Elemental Scientific Inc., Omaha, NE, USA), have greatly improved this process. The ongoing GEOTRACES program is providing a wealth of information on the distribution of Fe and other trace metals in the oceans [12]. The ratios of the different iron isotopes can be used to determine the sources of iron in the ocean when the isotopes signatures from different potential origins vary [220]. A study of Fe isotopes in the North Atlantic revealed that up to 87% of the dissolved iron comes from the Saharan desert [220]. The Fe dynamics during a subtropical phytoplankton bloom were monitored using stable isotopes [221] and demonstrated differences in fractionation when comparing differently sized phytoplankton. Smaller 0.2–20 µm cells are isotopically lighter than the <20 µm cells, likely due to the faster turnover of iron by smaller cells.

Detailed information on the uptake of iron by laboratory cultures of cyanobacteria could be obtained by measuring the loss of iron pools in the growth medium and changes in isotope and their ratios in the medium and cells. In addition, the importance or interactions of other metals that may be involved in the acquisition of iron could be elucidated. However, the equipment necessary is specialized and the techniques are not trivial. Yet, they are ideal methods where funds allow this analysis. Cost per sample may not be as prohibitive when balanced against the cost of radioisotopes and their limited life span and the limited information radioisotopes provide in comparison. A stable isotope measurement would be especially useful where radioactive isotope use is not permitted. However, the choice of medium used in experiments would need careful consideration as any ligands such as EDTA added to medium to regulate iron availability to cyanobacteria may compromise Fe measurements due to the binding of added spike metals during the analysis.

#### 6.6.3. Analysis of the Iron Oxidation State and Coordination Environment

The biological uptake of iron can lead to changes in the speciation of iron in the medium, e.g., the reduction of inorganically or organically complexed iron and subsequent incorporation into cell material. Although these changes can be hard to pinpoint, the oxidation state and coordination environment of iron in particles can be analyzed using x-ray absorption near the edge structure (XANES) and X-ray imaging [222,223,224]. With these techniques, it is possible to distinguish phases containing Fe(II) from Fe(III), and simultaneously discern variations in the coordination environment for each of the oxidation states. Employing this type of analysis in a culturing experiment will allow the identification of some speciation changes related to the growth and possible iron limitation in the culture.

In their 2012 study, von der Heyden et al. classified mineral phases into five broad classes, using identification of the Fe L3-edge by XANES analysis. They noted that natural samples, as well as samples from laboratory-grown cultures, are not as chemically pure or crystalline as one could wish, and these issues may complicate the analysis of generated spectra [225]. Furthermore, in their 2014 study, von der Heyden et al. identified common colloidal fractions of iron in natural waters by using scanning transmission X-ray microscopy (STXM) at the Fe L-edge and C K-edge as well as investigating the iron pools’ associations with natural organic molecules. They showed that Fe(II)-rich phases with a high degree of chemical heterogeneity were found in a range of different aquatic regimes, and that strong associations between these iron-rich particles and organic carbon suggests microbial preservation of Fe(II) [226]. This novel information regarding iron transformations occurring in cultures and natural samples demonstrates the benefits of these powerful characterization techniques. It is important to keep in mind, however, that chemically and structurally similar Fe phases will be difficult to distinguish using these techniques [226,227]. Such methods can be used to characterize the changes in inorganic iron present in a culture and provide information on whether the cultured organisms are able to alter the properties of added iron, or to determine the environments of trace metals within the cells themselves, as done, e.g., on Cu in *Anabaena flosaquae* [228].

## 7. Future Trends

One of the most important objectives for future cyanobacterial research should be interdisciplinary collaborations between scientists, which will provide complementary information, leading to increased speed of scientific discovery and progress. To achieve this, it is important that these collaborations are established at the experimental planning stages. An early and comprehensive understanding of what other research fields may contribute will enable scientists to more efficiently plan multi-faceted experiments, capable of providing comprehensive data sets. This requires scientists to communicate effectively across interdisciplinary boundaries and overcoming these boundaries will undoubtedly take time and effort in the initial stages. As mentioned in the introduction, we believe that further collaboration between biologists, chemists, and oceanographers offer unique insights into the role iron plays in the marine environment.

While we control drivers such as temperature, pH, and nutrient supply in the laboratory to elucidate the role of iron, none of these drivers of cyanobacterial growth are changing in isolation in the natural environment. Climate change, due to anthropogenic CO_2_ emissions, is causing massive global variations in parameters such as temperature and ocean pH [229]. Increased CO_2_ in the atmosphere has already caused a decrease in ocean pH by 0.1 [230] and is expected to decrease a further 0.3 units by 2100. The change in ocean chemistry may have large effects on the complexation and, thus, availability of trace metals [6]. Changes in pH may alter the structure of phytoplankton communities, resulting in changes in dissolved organic matter (DOM) release [231] causing decreased complexation of iron with the simultaneous increase in inorganic iron concentrations [232], which also depends on the original iron sources available. Increased anthropogenic activity and pollution are additional factors that also need to be considered.

A way forward in dealing with all this change would be well-designed multi-driver experiments, as they will give clearer answers to complex questions regarding modulations and interactions in the changing ocean [233]. However, the acquired data can be difficult to interpret, as it is not a trivial task to assign specific observed effects to specific stressors. Thus, a multiple driver experiment should seldom be the initial go-to as a new experimental series is set up. Simple, single-driver experiments can provide important background information, which will, in turn, make it easier to design multiple driver experiments [233]. Simpler pilot studies may not only reveal logistical challenges of multi-driver experiments but also help provide clearer results when observed effects of multi-stressor experiments are to be interpreted. Such pilot studies will also be of importance when developing new methodology, and especially when further improving existing methods.

A further complicating issue in understanding the role of iron in marine environments is the interactions between different strains and species of microorganisms. Axenic cultures often used in laboratory experiments are far from the natural state of microbial communities in the ocean. Therefore, co-culturing of species may be particularly interesting for studying the influences of single or multiple drivers. These experiments allow for complex community studies, using different classes of phytoplankton species, while simultaneously following processes on the physical and chemical level. This can be done using natural phytoplankton communities from the field either in mesocosm or lab studies, or by creating artificial communities based on the combination of different lab grown axenic cultures. The latter option allows for longer incubation times and better control of important parameters such as iron supply.

Since multiple driver and multiple species experiments are time-intensive, they require more physical lab space and often an array of instrumentation and analytical facilities. They further highlight the need for interdisciplinary collaborations. At the same time, monitoring and recording in situ changes as they happen also provides critical information. These observations should be the basis for further laboratory-based and in-situ effect studies to elucidate how any changes will affect ocean biogeochemistry as well as the biological communities living within and are connected to the global oceans. This again highlights the need for a consolidated effort, where methods are held to the highest standards, evaluated continuously, and, thus, produce results that are reproducible across different laboratories and research groups.

However, future ocean changes are likely to vary from region to region [234]. Thus, a concerted research effort is required to evaluate how changing parameters will affect the global ocean’s complex microbial communities. Collaborative studies with consistent, defined methods such as that of Boyd et al. [235] may be a way forward. Designing experiments that consolidate several of these stressors in different scenarios are imperative to understand the changes the planet is currently facing. No organism is facing the challenges of climate-related change alone, just as no single stressor is independent from other stressors. Combining our strengths and knowledge will make a difference.

## Supplementary Materials

The following are available online at https://www.mdpi.com/2076-2607/8/12/1889/s1.
