# Supplementary material for: From the Ocean to the Lab—Assessing Iron Limitation in Cyanobacteria: An Interface Paper
Source: Microorganisms. 2020 Nov 29;8(12):1889. doi: 10.3390/microorganisms8121889 (PMC7760322; doi:10.3390/microorganisms8121889)
Supplement: Supplementary file 1 [file microorganisms-08-01889-s001.zip › Technical manual.docx]

**Technical manual**

# Contents

[Introduction 1](#_Toc2340895)

[Cleaning of plastic consumables 1](#_Toc2340896)

[Preparation of Aquil 1](#_Toc2340897)

[Preparation of base medium 2](#_Toc2340898)

[Preparation of metal stock 3](#_Toc2340899)

[Preparation of vitamin stock 3](#_Toc2340900)

[Preparation of iron stock 4](#_Toc2340901)

[Finalize Aquil medium 4](#_Toc2340902)

[Preparation of Chelex-100 slurry 4](#_Toc2340903)

[Preparation of oxalate cell wash 5](#_Toc2340904)

[Preparation of NaCl rinse 5](#_Toc2340905)

[Sampling procedures 5](#_Toc2340906)

[Biological sampling 5](#_Toc2340907)

[Iron concentrations 6](#_Toc2340908)

[References 8](#_Toc2340909)

# Introduction

This technical manual combines different techniques presented in the review “From the ocean to the lab - assessing iron limitation in cyanobacteria: An interdisciplinary review” in order to conduct trace-metal free, sterile research with cyanobacteria, especially *Synechococcus* sp. PCC 7002, in a laboratory setting. The manual provides step by step instructions for cleaning consumables and preparation of the different chemicals and media used and concludes with an example of an effective sampling procedure. Workflows in Figure 2 depicte the several experimental procedures described in the aforementioned review.

# Cleaning of plastic consumables

All experimental procedures should be carried out using plasticware instead of glassware, this includes culture vessels, filters, sampling vials and tubing. All plastic ware should be cleaned in according to trace metal clean procedures [1,2]. Different plastics have different properties, and we suggest using recommendations from GEOTRACES (2017), e.g. Low-Density Polyethylene (LDPE) or High-Density Polyethylene (HPDE) for storage of samples, and [3], e.g. polycarbonate for growth experiments.

1. Immerse bottles in 5% detergent bath for one week
2. Rinse 5x with milli-Q (MQ) water, or more until no trace of detergent
3. Immerse bottles in 6M HCl (analytical grade) bath for 2 weeks
4. Rinse 3x with MQ water
5. Immerse in 3M analytical grade HNO_3_ bath for 2 weeks
6. Rinse 3x with MQ water
7. In a clean lab or clean air flow hood: Fill with MQ water and acidify to pH 2 with UP (Ultra Pure) HNO_3_ (12M) (1 mL per 1L MQ water) or with quartz distilled HCl to avoid yellowing of polycarbonate,
8. Double bag, seal and store inside large plastic bag within plastic box until use

Sterile pipette tips are rinsed with 10% quartz distilled HCl and then 3x with sterile MQ water immediately prior to use. This method is also used for acid washing filters.

# Preparation of Aquil

Aquil is an artificial seawater medium used for laboratory research. The original recipe was published in 1979 by Morel*, et al.* [4]. The recipe presented here is slightly modified to enable faster growth of *Synechococcus* sp. PCC 7002 under low-temperature conditions. Changes are indicated with an asterisk in the tables. This recipe is calculated to yield 1 L Aquil. Iron stock solutions should be prepared separately from other trace metals so that concentrations can be varied.

The recipe consists of three main tasks; preparing the base medium containing salts and nutrients, preparing the metal stock and preparing the vitamin stock.

## Preparation of base medium

1. Prepare anhydrous salt solution in 600 mL MQ water (table 1)

Table 1: Anhydrous salts

| **Anhydrous salts** | **[g]** | **Final concentration (M)** |
| --- | --- | --- |
| NaCl | 24.54 | 4.20E-01 |
| Na_2_SO_4_ | 4.09 | 2.88E-02 |
| KCl | 0.70 | 9.39E-03 |
| NaHCO_3_ | 0.20 | 2.38E-03 |
| KBr | 0.10 | 8.40E-04 |
| H_3_BO_3_ | 0.003 | 4.85E-05 |
| NaF | 0.003 | 7.15E-05 |

1. Prepare and dissolve hydrous salts in 300 mL MQ water, starting from the top of the table (table 2)

Table 2: Hydrous salts

| **Hydrous salts** | **[g]** | **Final concentration (M)** |
| --- | --- | --- |
| MgCl_2_x6H_2_O | 11.10 | 5.45E-02 |
| CaCl_2_x2H_2_O | 1.54 | 1.05E-02 |
| SrCl_2_x6H_2_O | 0.017 | 6.38E-05 |

1. Combine the 600 mL anhydrous and the 300mL hydrous salt solution into salt solution
2. Clean salt solution with the chelator Chelex-100 after following cleaning procedure detailed below [5]. This can be done by running the alt solution through a column packed with Chelex, or by the following procedure
   1. Add 1 mL Chelex slurry per 1 L Aquil. (see recipe for Chelex slurry below)
   2. Leave medium on shaker for 24 hrs
   3. Remove Chelex through trace metal clean filtration columns

NB! An alternative to Chelex-100 is Toyopearl (Tosoh) which avoids the long purification process needed for Chelex, but a longer residence time of the salt solution is required as the material is denser.

1. Sterilize salt solution by microwaving for subsequently 3, 2, 3 and 2 minutes in a 700W microwave. Bottles should be inverted for 30 seconds in between heating cycles. Allow to cool for 24 hours before further manipulations.
2. Prepare 1 L for each of the three major nutrient stock solutions (Table 3).

Table 3: Major nutrient stock solutions

| **Major nutrients** | **Stock**  **[g/L H_2_O]** | **mL**  **per L Aquil** | **Final concentration (M)** |
| --- | --- | --- | --- |
| NaH_2_PO_4_xH_2_O | 13.80 | 10 | 1.00E-03* |
| NaNO_3_ | 85.00 | 10 | 1.00E-02* |
| Na_2_SiO_3_x9H_2_O | 28.40 | 1 | 1.00E-04 |

* Concentrations increased compared to original Aquil recipe [3,4] in order to avoid nutrient limitation, based on previous studies using variations of medium A [6,7].

1. Chelex and subsequently filter-sterilize nutrient solutions using 0.2 µm acid washed filters and syringes (for filters, use acid washing procedure as for pipette tips mentioned above). If possible, use fully plastic syringes to avoid potential contamination from rubber plungers
2. Add chelexed and filter sterilized nutrient solutions to the salt solution

## Preparation of metal stock

1. Dissolve 2.92g EDTA in 950 mL MQ water for metal stock solution.
2. Add metals to EDTA solution (Table 4)

Table 4: Metal/ metalloids for EDTA solution

| **Metal/metalloids** | **[g]** | **Final concentration (M)** |
| --- | --- | --- |
| ZnSo_4_x7H_2_O | 0.023 | 7.97E-08 |
| MnCl_2_x4H_2_O | 0.024 | 1.21E-07 |
| CoCl_2_x&H_2_O | 0.012 | 5.03E-08 |
| Na_2_MoO_4_x2H_2_O | 0.0242 | 1.00E-07 |

1. Prepare two individual stock metal solutions of 1 L each (Table 5).

Table 5: Stock metal solutions

| **Metal** | **Stock**  **[g/L H_2_O]** | **[mL] used in EDTA-solution** | **Final concentration (M)** |
| --- | --- | --- | --- |
| CuSO_4_x5H_2_O | 4.9 | 1 | 1.96E-08 |
| Na_2_SeO_3_ | 1.9 | 1 | 1.00E-08 |

1. Add 1 mL of each stock metal solution to EDTA-metal solution
2. Bring EDTA-metal solution to 1 L
3. Filter sterilize EDTA-metal stock solution using 0.2 µm acid washed filters and syringes
4. Add EDTA-metal stock to medium

## Preparation of vitamin stock

1. Prepare 100 mL of vitamin stock solution (Table 6)

Table 6: Vitamin stock solution

| **Vitamin** | **Stock**  **[g/L H_2_O]** | **µL per L Aquil** | **Final concentration (M)** |
| --- | --- | --- | --- |
| Cyanocobalamin (Vit B12) | 0.005 | 100 | 3.70E-10 |

* Original Aquil recipe includes biotin (Vit. H) and Thiamine (Vit B1), which is not required for the growth of *Synechococcus* sp. PCC 7002. For other strains, this should be considered.

1. Filter-sterilize vitamin stock solution using 0.2 µm acid washed filters and syringes
2. Add 100µL vitamin stock to medium

## Preparation of iron stock

Iron stocks will differ according to experimental setup, but with low concentrations it may be preferable to use a pre-stock before preparing a final stock. An example of iron stocks is given in table 7.

1. Prepare iron stock solution by first preparing a pre-stock, which is used to prepare the final stock (Table 7). All pre-stock and final stock volumes in table are 100 mL.

Table 7: Iron stock solutions

| **Iron source** | **[g/100 mL]** | **Pre-stock**  **[M]** | **Pre-stock addition**  **[mL/100 mL]** | **Final stock**  **[M]** | **Final Concentration**  **[nM]** | **Stock**  **addition [uL]** |
| --- | --- | --- | --- | --- | --- | --- |
| FeCl_3_ x 6H_2_O | 2.70 | 0.1 | 1 | 0.001 | 50 | 50 |
| (NH_4_)_2_Fe(SO_4_)_2_ x 6H_2_O | 3.92 | 0.1 | 1 | 0.001 | 50 | 50 |
| FeO(OH) | 0.89 | 0.1 | 1 | 0.001 | 50 | 50 |

1. Filter sterilize iron stock solution using 0.2 µm acid washed filters and syringes
2. Add 50 µL of the required iron stock to medium

## Finalize Aquil medium

1. Bring medium to 1 L using MQ water
2. Add cell culture. Dilution will depend on density of starter culture and experimental conditions

# Preparation of Chelex-100 slurry

Initial approximate metal contamination of the salt solution should be determined by HR-ICP-MS analysis to calculate Chelex mass needed for efficient metal removal from the medium. If using Chelex-packed columns, this step is skipped, but metals in cleaned salt solution should be checked periodically.

- 1. Chelex mass = (mass of metals/Chelex resin wet capacity)*density of resin.
  2. Mass of metals = (total average mass of metals per volume [mg/L])/average equivalence
  3. Average equivalence = (Average molecular weight of metals/average valence)
  4. Wet capacity of Chelex = 0.40 meq/mL
  5. Density of Chelex resin = 0.65 g/mL

The above formulae [5] are used to determine the amount of Chelex required to remove metal present in the salt solution. The Chelex must be purified according to Price*, et al.* [8] before being added to the salt solution in the correct ratio. This purification procedure removes soluble metal binding ligands which may leach from the resin and compete with EDTA for metal complexation in the final medium.

# Preparation of oxalate cell wash

Oxalate wash and NaCl rinse are both used for determining intracellular iron concentrations mentioned under sampling procedures. Oxalate wash and NaCl rinse is prepared according to Hassler and Schoemann [9] and Tang and Morel [10].

1. Prepare 1L oxalate wash solution (table 8)

Table 7: Oxalate wash solution

| **Chemical** | **Final concentration [mM]** | **Amount [g/L]** |
| --- | --- | --- |
| NaCl | 100 | 5.844 |
| KCl | 10 | 0.7456 |
| Disodium oxalate | 100 | 13.4 |
| Disodium EDTA | 50 | 18.612 |

## Preparation of NaCl rinse

1. Prepare NaCl rinse solution (Table 9)

Table 8: Concentrations NaCl rinse

| **Chemical** | **Concentration [M]** | **Amount [g/L]** |
| --- | --- | --- |
| NaCl | 0.6 | 35.04 |
| NaHCO_3_ | 0.0024 | 0.20 |

1. Clean NaCl rinse solution with Chelex as described above for Aquil.

# Sampling procedures

All sampling vials and vessels should be twice rinsed with sample prior to final sampling. All acidification procedures use conc. UP HNO_3_ or quartz distilled HCl, to pH <2. Certified reference materials such as NASS-7 should be run regularly as part of the HR-ICP-MS analysis, including all preparation steps, such as filtering and pre-concentration.

## Biological sampling

1. Optical density at 730 nm (OD_730_) from culture and abiotic control
   1. Remove 3x1 mL subsamples at allotted times and transfer to cuvettes
   2. Measure OD_730_within 1 hour with a suitable spectrophotometer, using Aquil medium as a blanc
2. 77K fluorescence emission spectroscopy
   1. Remove 3x1 mL subsamples at allotted times
   2. Dilute samples to a maximum of 2µg/mL chlorophyll *a* in Aquil medium
   3. Transfer samples to sample glass tubes (inner diameter 2mm, outer diameter 5mm)
   4. Freeze samples in liquid nitrogen at -196°C (77K) until further analysis
   5. Record emission spectra at e.g. 440 nm and 580 nm excitation, for example with a setup described previously [11]
3. pH

* For monitoring purposes only. For accurate measurements of pH in seawater media, a more stringent procedure should be followed [12].

- 1. Remove 3x2 mL subsample
  2. Measure pH

## Iron concentrations

The following sampling procedures include sampling for direct analysis, and for preconcentration using SeaFAST. Only one of these may be necessary depending on the iron concentration used. This should be evaluated on an individual experimental basis. Samples should be analyzed during experiment if possible, to be able to monitor any potential contamination.

1. Total Fe for HR-ICP-MS from culture and abiotic control
   1. Remove 3x5 mL subsamples into acid washed tube
   2. Acidify 5 mL subsamples pH < 1.7
   3. Remove 25 mL sample into acid washed tube – for SeaFAST preconcentration
   4. Acidify 25 mL subsamples (NB! To break all EDTA complexes before preconcentration, it may be necessary to UV radiate samples)
   5. Blanks are prepared using acidified MQ water
2. Dissolved Fe for ICP-MS from culture and abiotic control
   1. Remove 3x5 mL subsamples and filter through 0.2 µm acid washed syringe and filter into acid washed tube (rinse syringe, filter and tube with sample before collecting final sample)
   2. Acidify 5 mL subsamples
   3. Remove 3x25 mL subsamples into acid washed tube by filtering through 0.2 µm syringe filter using syringe – for SeaFAST preconcentration
   4. Acidify 25 mL subsamples
   5. Blanks are prepared using acidified MQ water
3. Particulate Fe for HR-ICP-MS from culture
   1. Remove appropriate subsample volumes (depending on culture density) in triplicate
   2. Pass subsamples through 45 mm 0.2 µm polycarbonate filter inside Nalgene filter system (Thermo Scientific) until pigment is visible on the filter. Note volume of subsample filtered
   3. Transfer filter to acid cleaned petri dish. Seal using tape
   4. Freeze filter until ready for digestion by Ultra-Clave before HR-ICP-MS analysis
   5. Samples should be taken from abiotic control at regular interval to control for particulate contamination
   6. Blanks are prepared by running 25 mL MQ through the filter
4. Intracellular Fe for HR-ICP-MS
   1. Remove appropriate subsample volumes (depending on culture density) in triplicate
   2. Pass subsamples through 45 mm 0.2 µm polycarbonate filter inside Nalgene filter system (Thermo Scientific) until pigment is visible on the filter. Note volume of subsample filtered
   3. When approx. 5 mL of subsample remains, remove vacuum and add 10 mL oxalate wash to the filter containing the subsample
   4. Leave oxalate wash on filter for 5 minutes
   5. Re-apply vacuum
   6. Rinse filter with ten 1.5 mL aliquots of chelexed NaCl rinse solution
   7. Transfer filter to acid cleaned petri dish. Seal using tape
   8. Freeze filter until ready for digestion by Ultra-Clave before HR-ICP-MS analysis
   9. Samples should be taken from abiotic control at regular interval to control for any potential particulate contamination (analyzed during experiment if possible)
   10. Blanks are prepared by running 25 mL MQ water through the filter, with subsequent treatment by oxalate and NaCl rinse solutions
5. Fe(II) on FIA-CL
   1. A five-step calibration curve is run before every sampling session
   2. Subsamples must be collected from the culture immediately prior to analysis
   3. 5-6 parallels should be analyzed for each subsample, to account for oxidation during the sampling process
   4. Subsample is filtered through 0.2 µm acid washed syringe and membrane filters before introduction to the FIA system
   5. Luminol reagent should be prepared and dark aged for at least 24 hours prior to use, according to Croot and Laan (2002)
   6. Primary, secondary and tertiary iron standards are prepared according to Croot and Laan [13]. Concentrations of working standard solutions should be chosen according to initial iron concentration and source and can be identified by running pre-experimental tests
   7. Fe(II) blanks are prepared by dark aging Aquil medium.

# References

1. Achterberg, E.P.; Holland, T.W.; Bowie, A.R.; Fauzi, R.; Mantoura, C.; Worsfold, P.J. Determination of iron in seawater. *Anal Chim Acta* **2001**, *442*, 1-14, doi:Doi 10.1016/S0003-2670(01)01091-1.

2. GEOTRACES. Cruice and methods manual (Cookbook). Availabe online: (accessed on

3. Sunda, W.G.; Price, N.M.; Morel, F.M.M. Trace metal ion buffers and their use in culture studies. In *Algal Culturing Techniques*, Andersen, R.A., Ed. Elsevier Academic Press: London, UK, 2005.

4. Morel, F.M.M.; Rueter, J.G.; Anderson, D.M.; Guillard, R.R.L. Aquil - Chemically Defined Phytoplankton Culture-Medium for Trace-Metal Studies. *J Phycol* **1979**, *15*, 135-141, doi:DOI 10.1111/j.0022-3646.1979.00135.x.

5. Bio-Rad-Laboratories. Chelex 100 and Chelex 20 chelating ion exchange resin: Instruction manual. Availabe online: <http://www.bio-rad.com/webmaster/pdfs/9184_Chelex.PDF> (accessed on 20.01.2020).

6. Stevens Jr, S.E.; Patterson, C.O.P.; Myers, J. THE PRODUCTION OF HYDROGEN PEROXIDE BY BLUE-GREEN ALGAE: A SURVEY1. *J Phycol* **1973**, *9*, 427-430, doi:10.1111/j.1529-8817.1973.tb04116.x.

7. Ludwig, M.; Bryant, D.A. Transcription profiling of the model cyanobacterium Synechococcus sp strain PCC 7002 by Next-Gen (SOLiD (TM)) sequencing of cDNA. *Front Microbiol* **2011**, *2*, doi:ARTN 41 10.3389/fmicb.2011.00041.

8. Price, N.M.; Harrison, G.I.; Hering, J.G.; Hudson, R.J.; Nirel, P.M.V.; Palenik, B.; Morel, F.M.M. Preparation and Chemistry of the Artificial Algal Culture Medium Aquil. *Biological Oceanography* **1989**, *6*, 443-461, doi:10.1080/01965581.1988.10749544.

9. Hassler, C.S.; Schoemann, V. Discriminating between intra- and extracellular metals using chemical extractions: an update on the case of iron. *Limnol Oceanogr-Meth* **2009**, *7*, 479-489, doi:DOI 10.4319/lom.2009.7.479.

10. Tang, D.G.; Morel, F.M.M. Distinguishing between cellular and Fe-oxide-associated trace elements in phytoplankton. *Mar Chem* **2006**, *98*, 18-30, doi:10.1016/j.marchem.2005.06.003.

11. Lamb, J.J.; Rokke, G.; Hohmann-Marriott, M.F. Chlorophyll fluorescence emission spectroscopy of oxygenic organisms at 77 K. *Photosynthetica* **2018**, *56*, 105-124, doi:10.1007/s11099-018-0791-y.

12. Dickson, A.G. The measurement of sea water pH. *Mar Chem* **1993**, *44*, 131-142, doi:<https://doi.org/10.1016/0304-4203(93)90198-W>.

13. Croot, P.L.; Laan, P. Continuous shipboard determination of Fe(II) in polar waters using flow injection analysis with chemiluminescence detection. *Anal Chim Acta* **2002**, *466*, 261-273, doi:Doi 10.1016/S0003-2670(02)00596-2.
